# *Mycobacterium tuberculosis* RecG binds and unwinds model DNA substrates with a preference for Holliday junctions

**DOI:** 10.1099/mic.0.058693-0

**Published:** 2012-08

**Authors:** Ephrem Debebe Zegeye, Seetha V. Balasingham, Jon K. Laerdahl, Håvard Homberset, Tone Tønjum

**Affiliations:** 1 Centre for Molecular Biology and Neuroscience and Department of Microbiology, University of Oslo, Oslo, Norway; 2 Department of Microbiology, Oslo University Hospital (Rikshospitalet), Oslo, Norway; 3 Bioinformatics Core Facility, Department of Informatics, University of Oslo, Oslo, Norway

## Abstract

The RecG enzyme, a superfamily 2 helicase, is present in nearly all bacteria. Here we report for the first time that the *recG* gene is also present in the genomes of most vascular plants as well as in green algae, but is not found in other eukaryotes or archaea. The precise function of RecG is poorly understood, although ample evidence shows that it plays critical roles in DNA repair, recombination and replication. We further demonstrate that *Mycobacterium tuberculosis* RecG (RecG_Mtb_) DNA binding activity had a broad substrate specificity, whereas it only unwound branched-DNA substrates such as Holliday junctions (HJs), replication forks, D-loops and R-loops, with a strong preference for the HJ as a helicase substrate. In addition, RecG_Mtb_ preferentially bound relatively long (≥40 nt) ssDNA, exhibiting a higher affinity for the homopolymeric nucleotides poly(dT), poly(dG) and poly(dC) than for poly(dA). RecG_Mtb_ helicase activity was supported by hydrolysis of ATP or dATP in the presence of Mg^2+^, Mn^2+^, Cu^2+^ or Fe^2+^. Like its *Escherichia coli* orthologue, RecG_Mtb_ is also a strictly DNA-dependent ATPase.

## Introduction

*Mycobacterium tuberculosis*, the aetiological agent of the re-emerging disease tuberculosis (TB), remains a global health threat, killing at least 1.5 million individuals every year (WHO, 2011). The emergence of extensively and extremely drug-resistant *M. tuberculosis* strains, coupled with the HIV/AIDS pandemic, has exacerbated the risk of TB resurgence, underlining the urgent need to develop interventions that halt the spread of this disease. *M. tuberculosis*, an intracellular human pathogen, successfully combats many host cell defence mechanisms, including genotoxic stress, using efficient DNA repair pathways that help to maintain its genome integrity, in spite of accumulating DNA damage during infection (Ambur *et al.*, 2009; Dos Vultos *et al.*, 2009; Gorna *et al.*, 2010; Mizrahi & Andersen, 1998; Stallings & Glickman, 2010). It is believed that these DNA repair pathways promote *M. tuberculosis* survival and increase its pathogenicity and virulence (Gorna *et al.*, 2010; Warner, 2010). Thus, in-depth characterization of the mechanisms that protect the *M. tuberculosis* genome and promote its virulence and/or capacity to develop drug resistance may lead to novel therapeutic targets and attenuate the increasing risk of global resurgence of TB.

*Escherichia coli* RecG, a 76 kDa monomeric helicase with a particular affinity for branched-DNA substrates such as replication forks, Holliday junctions (HJs), and D- and R-loops, has been shown to play roles in DNA repair, recombination and replication (Lloyd & Sharples, 1993; McGlynn *et al.*, 1997; McGlynn & Lloyd, 2000, 2001; Rudolph *et al.*, 2010b; Vincent *et al.*, 1996; Whitby & Lloyd, 1998). *E. coli* RecG is widely believed to promote regression of stalled replication forks, when fork progression is blocked by lesions in the DNA template strand, thereby facilitating repair or bypass of the lesion (McGlynn & Lloyd, 2001, 2002). *In vitro* studies suggest that RecG actively unwinds stalled replication forks, generating a four-way junction product that resembles an HJ (McGlynn & Lloyd, 2000, 2001; McGlynn *et al.*, 2001), and also promotes HJ branch migration (Müller & West, 1994; Whitby *et al.*, 1994). Structural characterization of a complex between *Thermotoga maritima* RecG and a forked-DNA substrate has revealed the mechanism by which RecG recognizes junctions (Singleton *et al.*, 2001). *E. coli* RecG inhibits inappropriate DNA amplification and aberrant chromosome segregation in cells exposed to UV irradiation (Rudolph *et al.*, 2009a, b), and is also essential in cells lacking 3′ ssDNA exonucleases to counteract PriA helicase-mediated DNA re-replication (Rudolph *et al.*, 2010a). Furthermore, a recent study has revealed that RecG promotes resolution of intermolecular recombination intermediates that are poorly recognized/resolved by RuvABC (Fonville *et al.*, 2010).

Strains with mutations in *recG* have been shown to exhibit complex and variable phenotypes, including transformation deficiency in *Neisseria meningitidis* (Sun *et al.*, 2005), growth defects and reduced radio-resistance in *Deinococcus radiodurans* (Wu *et al.*, 2009), and sensitivity to UV irradiation and oxidative stress in *Pseudomonas aeruginosa* (Ochsner *et al.*, 2000). *recG* mutation has also been suggested to be responsible for the susceptibility of *Staphylococcus aureus* to quinolone (Niga *et al.*, 1997) and of *E. coli* to bleomycin, metronidazole and ciprofloxacin (Kosa *et al.*, 2004; Tamae *et al.*, 2008).

In this study, we have characterized the recombinant RecG enzyme from *M. tuberculosis* H37Rv (RecG_Mtb_) for its DNA binding, unwinding and ATPase activities in order to delineate its potential roles in the DNA metabolism of *M. tuberculosis*.

## Methods

### 

#### Cloning the *M. tuberculosis recG* gene.

*M. tuberculosis* genomic DNA was isolated from a *M. tuberculosis* H37Rv (ATCC 25618) culture. The *M. tuberculosis recG* (Rv2973c) gene was PCR-amplified using a forward primer (5′-CGCATATGGCGTCGTTAAGCGATCGGCTC-3′) and reverse primer (5′-CGCTCGAGTCATGACTTATCTAAGTATTCGATGC-3′) which contained *Nde*I and *Xho*I restriction sites, respectively (underlined). The PCR product was digested with *Nde*I and *Xho*I and ligated into a similarly digested pET28b(+) vector (Novagen). The resulting construct, pET28b–recG with an N-terminal His_6_-tag, was then transformed into *E. coli* ER2566 (New England Biolabs; NEB). The nucleotide sequence of the construct was verified by DNA sequencing (ABI).

#### Overexpression and purification of the RecG_Mtb_ protein.

The recombinant RecG_Mtb_ protein was purified to homogeneity as follows. *E. coli* ER2566 harbouring pET28b–recG was inoculated into Luria–Bertani broth (Difco) supplemented with 50 µg kanamycin ml^−1^, 2.5 mM betaine hydrochloride and 0.5 M sorbitol, and grown at 37 °C to OD_600_ ~0.4. The culture was then transferred to 18 °C and induced at OD_600_ ~0.6 with 0.5 mM IPTG. After overnight growth, cells were harvested, lysed and purified using a nickel-nitrilotriacetic acid agarose column as described in the QIAexpressionist protocol for native purification of His_6_-tagged proteins from *E. coli* (Qiagen, 2003). β-Mercaptoethanol (5 mM) was added to the lysis, wash and elution buffers as indicated in the protocol. After elution from the column, the eluates containing RecG_Mtb_ were pooled and dialysed overnight against buffer comprising 50 mM NaH_2_PO_4_ (pH 7.5), 300 mM NaCl and 1 mM DTT. The N-terminal His_6_-tag was cleaved off using biotinylated thrombin (Novagen) following the manufacturer’s protocol. Further purification was carried out on a HiTrap Q HP column (GE Healthcare) after the buffer had been exchanged with 20 mM Bistris (pH 7.2), 100 mM NaCl and 1 mM DTT. Fractions containing pure RecG_Mtb_ were pooled and dialysed against storage buffer [20 mM Bistris (pH 7.5), 300 mM NaCl, 1 mM DTT, 20 % glycerol (w/v)] and stored at −80 °C until use. RecG_Mtb_ protein concentration was determined using the Bradford method (Bio-Rad) using BSA as standard.

#### Model DNA substrate preparation for DNA binding, unwinding and ATPase assays.

DNA substrates were prepared essentially as described by Brosh *et al.* (2006) with some modifications. Briefly, oligonucleotides were 5′ end-labelled using [γ-^32^P]ATP (PerkinElmer) and T4 PNK enzyme (NEB) for 1 h at 37 °C, followed by enzyme inactivation at 65 °C for 20 min. Unincorporated ATPs were removed using illustra MicroSpin G-25 columns (GE Healthcare). Labelled and unlabelled complementary oligonucleotides were mixed at a molar ratio of 1 : 2.5 in annealing buffer [40 mM Tris/HCl (pH 8.0), 50 mM NaCl], denatured at 95 °C for 5 min, and allowed to cool to room temperature for about 3 h. The annealed products were resolved on an 8 % non-denaturing polyacrylamide gel. The bands containing the completely annealed substrates were excised and DNA was eluted into buffer comprising 10 mM Tris/HCl (pH 8.0) and 0.5 mM EDTA by incubating overnight at 4 °C. The concentrations of the eluted DNA substrates were estimated as described by Brosh *et al.* (2006) and are given in moles of substrate molecules. For ATPase assays, DNA cofactors employed were prepared by annealing equimolar concentrations of complementary strands. Proper annealing of the prepared DNA cofactors was verified by resolving on a non-denaturing 10 % polyacrylamide gel and staining with SYBR Safe DNA Gel Stain (Invitrogen). The oligonucleotides used and the schematics of the model DNA substrates constructed are presented in Tables 1 and 2, respectively.

**Table 1.** List of oligonucleotides used to construct model DNA substrates

Oligonucleotide no.	Oligonucleotide name	Length (nt)	Sequence (5′–3′)	Reference or source
1	A	40	CGTGACATGCCGTGACTAGCTTTTTTTTTTTTTTTTTTTT	Biswas *et al.* (2009)
2	B	20	GCTAGTCACGGCATGTCACG	Biswas *et al.* (2009)
3	C	40	TTTTTTTTTTTTTTTTTTTTCGTGACATGCCGTGACTAGC	Biswas *et al.* (2009)
4	HJSO1	49	GACGCTGCCGAATTCTGGCTTGCTAGGACATCTTTGCCCACGTTGACCC	Lloyd & Sharples (1993)
5	HJSO2	50	TGGGTCAACGTGGGCAAAGATGTCCTAGCAATGTAATCGTCTATGACGTT	Lloyd & Sharples (1993)
6	HJSO3	51	CAACGTCATAGACGATTACATTGCTAGGACATGCTGTCTAGAGACTATCGA	Lloyd & Sharples (1993)
7	HJSO4	50	ATCGATAGTCTCTAGACAGCATGTCCTAGCAAGCCAGAATTCGGCAGCGT	Lloyd & Sharples (1993)
8	HJSO5	49	CGGGTCAACGTGGGCAAAGATGTCCTAGCAAGCCAGAATTCGGCAGCGT	This study
9	RFSO1	50	GTCGGATCCTCTAGACAGCTCCATGATCACTGGCACTGGTAGAATTCGGC	McGlynn & Lloyd (2001)
10	RFSO2	50	CAACGTCATAGACGATTACATTGCTACATGGAGCTGTCTAGAGGATCCGA	McGlynn & Lloyd (2001)
11	RFSO3	25	TAGCAATGTAATCGTCTATGACGTT	McGlynn & Lloyd (2001)
12	RFSO4	26	TGCCGAATTCTACCAGTGCCAGTGAT	McGlynn & Lloyd (2001)
13	DLO1	61	GGGTGAACCTGCAGGTGGGCGGCTGCTCATCGTAGGTTAGTTGGTAGAATTCGGCAGCGTC	McGlynn *et al.* (1997)
14	DLO2	61	GACGCTGCCGAATTCTACCAGTGCCTTGCTAGGACATCTTTGCCCACCTGCAGGTTCACCC	McGlynn *et al.* (1997)
15	DLO3	41	TAAGAGCAAGATGTTCTATAAAAGATGTCCTAGCAAGGCAC	McGlynn *et al.* (1997)
16	DLO4	41	AAAGATGTCCTAGCAAGGCACGATCGACCGGATATCTATGA	McGlynn *et al.* (1997)
17	DLO5	61	TATAGAACATCTTGCTCGTTTTCGAGCAAGATGTTCTATAAAAGATGTCCTAGCAAGGCAC	This study
18	DLO6	61	AAAGATGTCCTAGCAAGGCACGATCGACCGGATATCTACTTTTGTAGATATCCGGTCGATC	This study
19	DLO7	21	AAAGATGTCCTAGCAAGGCAC	McGlynn *et al.* (1997)
20*	2-Methyl RNA-1	41	UAAGAGCAAGAUGUUCUAUAAAAGAUGUCCUAGCAAGGCAC	This study
21*	2-Methyl RNA-2	41	AAAGAUGUCCUAGCAAGGCACGAUCGACCGGAUAUCUAUGA	This study
22	HJSO6	66	TGGGTCAACGTGGGCAAAGATGTCCTAGCAATGTAATCGTCTATGACGTTGTTTTTTTTTTTTTTT	This study

* 2′-*O*-Methyl-RNA.

**Table 2.**
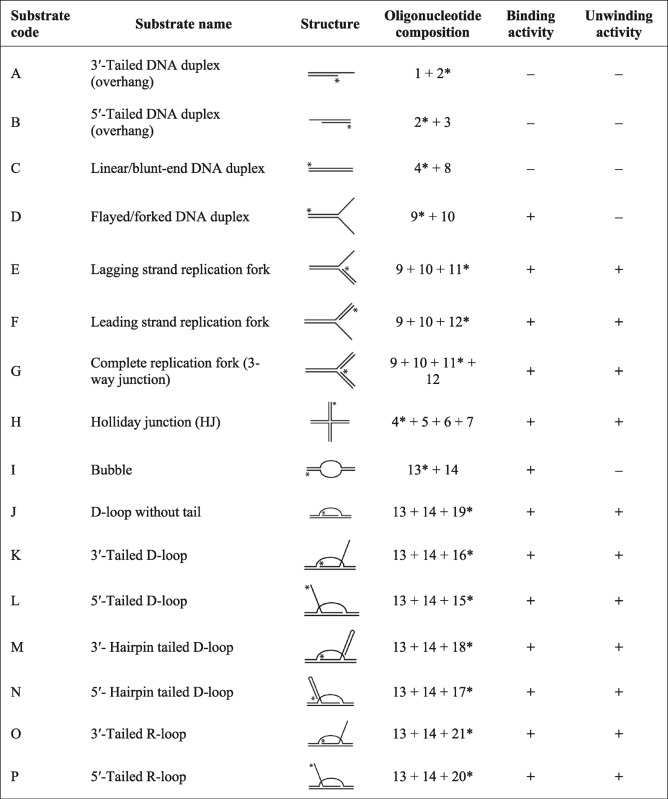
Summary of RecG_Mtb_ DNA binding and unwinding activity Minus and plus symbols indicate the absence or presence of RecG_Mtb_ activity on the indicated substrate, respectively.

* γ-^32^P-Labelled 5′ end.

#### DNA binding assays.

DNA binding assays were carried out as described by Whitby & Lloyd (1998) with some modifications. Assays were performed in reactions (20 µl) containing binding buffer [50 mM Tris/HCl (pH 8.0), 5 mM EDTA, 100 µg BSA ml^−1^, 6 % (w/v) glycerol, 2 mM DTT, 50 ng poly(dI-dC) µl^−1^ (Thermo Scientific)] and 0.1 nM of the indicated DNA substrate. Where indicated, poly(dI-dC) was omitted from the binding buffer. Reactions were initiated by adding indicated concentrations of RecG_Mtb_. After 15 min incubation on ice, 4 µl of 60 % (w/v) glycerol was added and immediately loaded onto a pre-cooled and pre-run (30 min) 4 % non-denaturing polyacrylamide gel (29 : 1). Electrophoresis was performed using low-ionic-strength buffer at 200 V for 5 min and at 160 V for an additional 85 min in an ice-water bath with buffer recirculation. Gels were dried, exposed to a phosphorimaging screen, visualized using a phosphorimager (Typhoon 9410, Amersham Biosciences) and quantified by ImageQuant TLv 2003.02 software (GE Healthcare).

#### Helicase assays.

Unless otherwise specified, all helicase assays were conducted in a 20 µl reaction containing helicase buffer [20 mM Tris/HCl (pH 7.5), 2 mM MgCl_2_, 2 mM ATP, 100 µg BSA ml^−1^, 2 mM DTT], 0.5 nM DNA substrate and the indicated concentrations of RecG_Mtb_. For divalent metal cofactor studies, MgCl_2_ in the aforementioned buffer was replaced by 2 mM of the indicated metal chloride. Similarly, in fuel preference studies, 2 mM of the tetrasodium salt of the indicated NTP/dNTP was used in place of ATP in the helicase buffer. Helicase reactions were initiated by adding RecG_Mtb_ and, after incubating at 37 °C for 30 min, were terminated with 10 µl of 3× helicase stop solution [50 mM EDTA, 40 % (w/v) glycerol, 0.9 % SDS, 0.1 % bromophenol blue, 0.1 % xylene cyanol] containing a 10-fold molar excess of trap oligonucleotide. For helicase time-course assays, the reaction was scaled up to 140 µl and RecG_Mtb_ was added into pre-incubated (3 min) reaction mixture at 37 °C. An aliquot (10 µl) of the reaction mixture was withdrawn at the indicated time points and mixed with 5 µl 3× helicase stop solution. All helicase reaction products were resolved by 10 % non-denaturing polyacrylamide (19 : 1) gel electrophoresis at 150 V for 2 h at room temperature using 1× Tris/borate-EDTA buffer. Gels were dried and analysed as described for DNA binding assays. The proportion of helicase substrate unwound (%) was calculated as described by Brosh *et al.* (2006).

#### ATPase assays.

RecG_Mtb_ ATP hydrolysis activity was monitored by TLC, as described by Kornberg *et al.* (1978) with some modifications. Briefly, RecG_Mtb_ was added to initiate a 10 µl reaction in ATPase buffer [20 mM Tris/HCl (pH 7.5), 2 mM MgCl_2_, 100 µg BSA ml^−1^, 25 µM cold ATP, 0.023 nM [γ-^32^P]ATP, 2 mM DTT] and the indicated DNA cofactor. The reaction was incubated at 37 °C for the indicated times and terminated by adding 5 µl 0.5 M EDTA (pH 8.0). Samples (2 µl) were spotted onto TLC plates (Cellulose PEI F, Merck) at 1.5 cm intervals and resolved using a solution containing 1 M formic acid and 0.5 M LiCl. The TLC plates were air-dried, exposed to a phosphorimaging screen, imaged and quantified as described above for the DNA binding assays. The proportion of hydrolysed ATP (%) was calculated as {counts for γ-^32^P_i_/(counts for γ-^32^P_i_+counts for [γ-^32^P]ATP)}×100. The values obtained from samples lacking RecG_Mtb_ were subtracted from the samples containing RecG_Mtb_ to account for background ATP hydrolysis. An unpaired Student’s *t* test was used to determine statistical significance.

To determine the steady-state kinetic parameters of ATP hydrolysis, a 20 µl reaction was set up with ATPase buffer [20 mM Tris/HCl (pH 7.5), 4 mM MgCl_2_, 100 µg BSA ml^−1^, cold ATP, 0.023 nM [γ-^32^P]ATP, 2 mM DTT], 125 ng plasmid DNA (pET28b) µl^−1^ and 150 nM RecG_Mtb_. The cold ATP concentration was varied between 100 and 800 µM. The reactions were incubated at 37 °C for 10 min and quenched with 10 µl 0.5 M EDTA (pH 8.0). The concentration of hot ATP was negligible and thus not considered in the calculations. The velocity data points versus cold ATP concentrations were non-linearly fitted to Michaelis–Menten and Hill equations using Prism 5 software (GraphPad).

## Results

### RecG is conserved in bacteria and is present in vascular plants

**Fig. 1.**
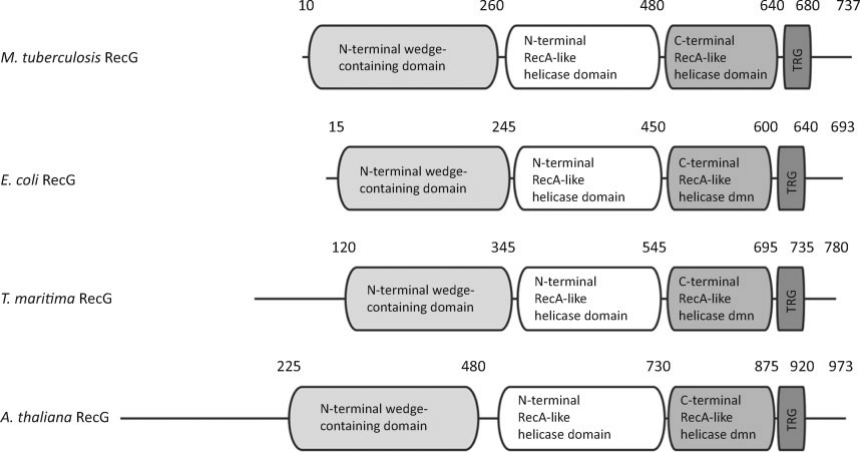
RecG, with its unique wedge-containing domain, is found in bacteria and plants. Domain organization showing the N-terminal wedge-containing domain that is unique to the RecG helicase family, the N- and C-terminal RecA-like helicase domains, and the C-terminal TRG motif for RecG from the bacteria *M. tuberculosis*, *E. coli* and *T. maritima*, and the vascular plant *Arabidopsis thaliana*. Numbers indicate approximate domain boundaries.

The full-length *E. coli* RecG protein sequence was used as a query to search NCBI protein sequence databases (Sayers *et al.*, 2012) for conserved homologues in bacteria, archaea, and plants and other eukaryotes. Homologues of *recG* were found to be present in the genomes of most bacteria, except Chlamydiae and Mollicutes, as reported earlier (Rocha *et al.*, 2005; Sharples *et al.*, 1999). However, *recG* homologues were not found in any of the >90 archaeal genomes in the current version of the database (Sayers *et al.*, 2012). Notably, full-length *recG* was also present in the genomes of most vascular plants, including *Arabidopsis thaliana*, the castor oil plant (*Ricinus communis*), common grape vine (*Vitis vinifera*), California poplar (*Populus trichocarpa*) and the lycophyte *Selaginella moellendorffii*, as well as in green algae such as *Ostreococcus tauri* and *Nannochloris bacillaris*. The *recG* gene was not detected in eukaryote species outside the kingdom Plantae. The plant genes are localized to the nuclear genomes and do not appear to be recently acquired from bacteria. For example, *A. thaliana* (Arabidopsis Genome Initiative, 2000) and *V. vinifera* (Jaillon *et al.*, 2007) have intron-rich *recG* genes encoded on chromosomes 2 and 1, respectively, with all 16 introns shared between the two species. The RecG homologues, including RecG_Mtb_, have two RecA-like helicase domains, an N-terminal wedge-containing domain and a C-terminal TRG (translocation in RecG) motif (Mahdi *et al.*, 2003) (Fig. 1).

### RecG_Mtb_ binds a wide variety of DNA substrates

**Fig. 2.**
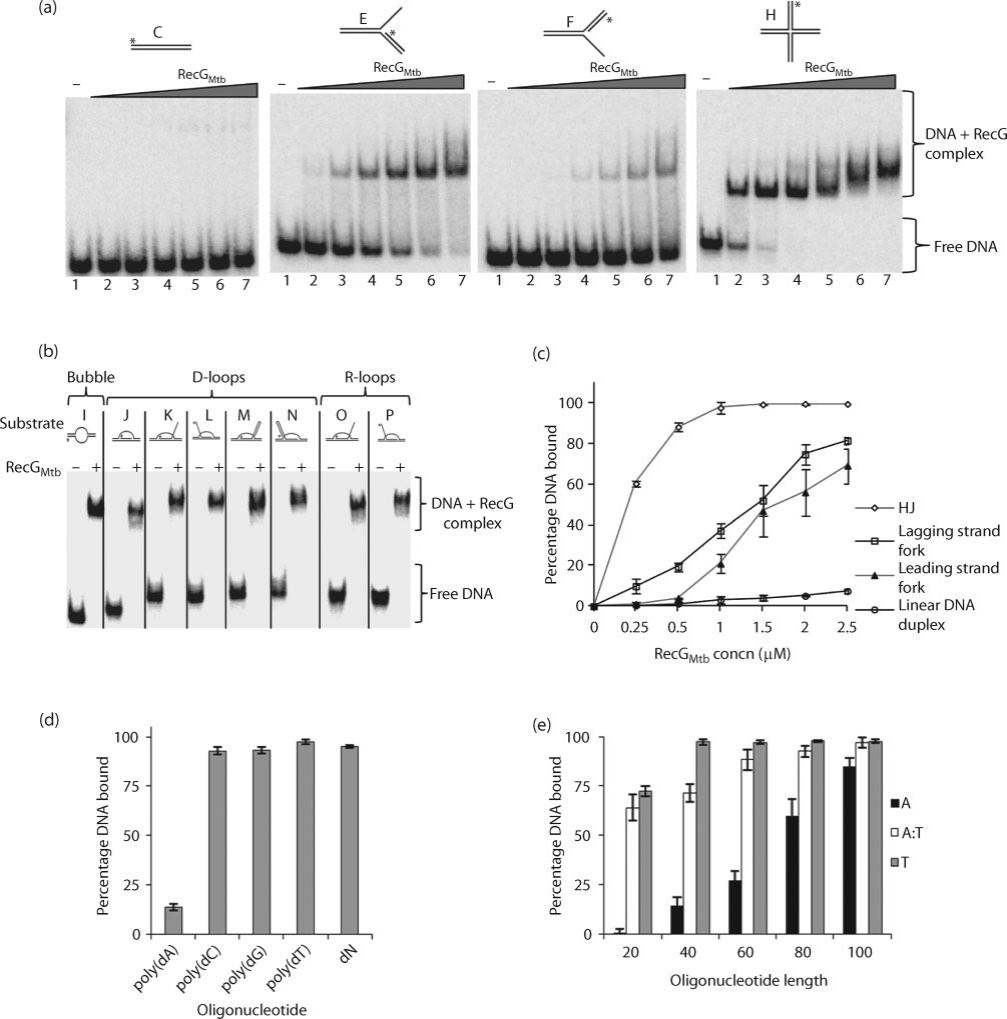
RecG_Mtb_ DNA binding specificity. (a) Titrations of the DNA binding activity of RecG_Mtb_ using 0.25, 0.5, 1.0, 1.5, 2.0 and 2.5 µM RecG_Mtb_ (lanes 2–7, respectively) and 0.1 nM linear DNA duplex (substrate C), lagging strand replication fork (substrate E), leading strand replication fork (substrate F) and HJ (substrate H). (b) Gel shift assay on a bubble and a variety of D- and R-loop substrates in the presence of 2 µM RecG_Mtb_. Lanes: (−), absence of RecG_Mtb_; (+), presence of RecG_Mtb_. (c) Quantification of the gel images in (a). (d) Shift assays using 0.1 nM of 40 nt poly(dA), poly(dC), poly(dG), poly(dT) and random nucleotide (dN), and 1 µM RecG_Mtb_ in the absence of poly(dI-dC). (e) ssDNA and dsDNA binding activity of RecG_Mtb_ using 0.1 nM poly(dA) (A), poly(dT) (T) and poly(dA : dT) (A : T) of increasing length and 1 µM RecG_Mtb_ in the absence of poly(dI-dC). Data presented in (c–e) are means±sd from at least three independent experiments.

RecG_Mtb_ bound to a wide variety of model DNA substrates, including partial and complete replication forks, HJ, bubble, and D- and R-loop substrates (Fig. 2a, b, Table 2), with the highest affinity for HJs (Fig. 2c). In contrast, the affinity of RecG_Mtb_ for a linear DNA duplex (49 bp) was very low, and it did not bind DNA substrates containing 20 nt 5′ or 3′ overhangs, in the presence of poly(dI-dC) competitor (Fig. 2a, c, Table 2). When we analysed the binding affinity of RecG_Mtb_ to homopolymeric nucleotides (40 nt) in the absence of poly(dI-dC) competitor, poly(dA) showed very weak binding compared with poly(dC), poly(dG), poly(dT) and random nucleotides (dN) (Fig. 2d). However, the binding affinity of RecG_Mtb_ to poly(dA) appeared to increase with increasing length of nucleotides as for poly(dT) and poly(dA : dT), yet with less stable protein–DNA complexes (Fig. 2e; data not shown). The binding activity of RecG_Mtb_ was not influenced by the presence or absence of ATP, ADP or ATPγS (see Fig. S1 available with the online version of this paper).

### RecG_Mtb_ unwinds DNA replication forks, D-loops, R-loops and HJ substrates

**Fig. 3.**
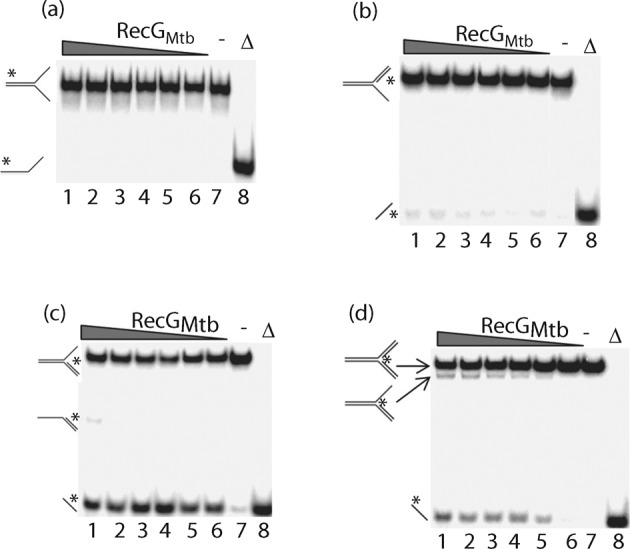
RecG_Mtb_ unwinds nascent DNA strands from partial and complete replication fork substrates. Titration of helicase activity using 0.5 nM flayed DNA duplex (a), leading strand replication fork (b), lagging strand replication fork (c) and complete replication fork (d) substrates, and 250, 200, 150, 100, 50 and 25 nM RecG_Mtb_ (lanes 1–6, respectively). Lanes: (−), reaction lacking RecG_Mtb_; (Δ), heat-denatured substrate.

The unwinding activity of RecG_Mtb_ was examined using a variety of DNA substrates, including flayed DNA duplex, and lagging, leading and complete replication fork substrates. RecG_Mtb_ did not exert any unwinding activity on flayed DNA duplex, but demonstrated weak and strong unwinding activities on leading and lagging strand replication forks, respectively (Fig. 3a–c). Moreover, RecG_Mtb_ unwound both strands of a complete replication fork substrate (Fig. 3d). These results suggested that RecG_Mtb_, like *E. coli* RecG, requires more than one duplex arm to unwind a three-way junction (Whitby & Lloyd, 1998). Interestingly, both RecG_Mtb_ and *E. coli* RecG (McGlynn & Lloyd, 2001) unwound the lagging strand replication fork more efficiently than the leading strand replication fork substrate (Fig. 3b, c; see also Fig. 5c).

**Fig. 4.**
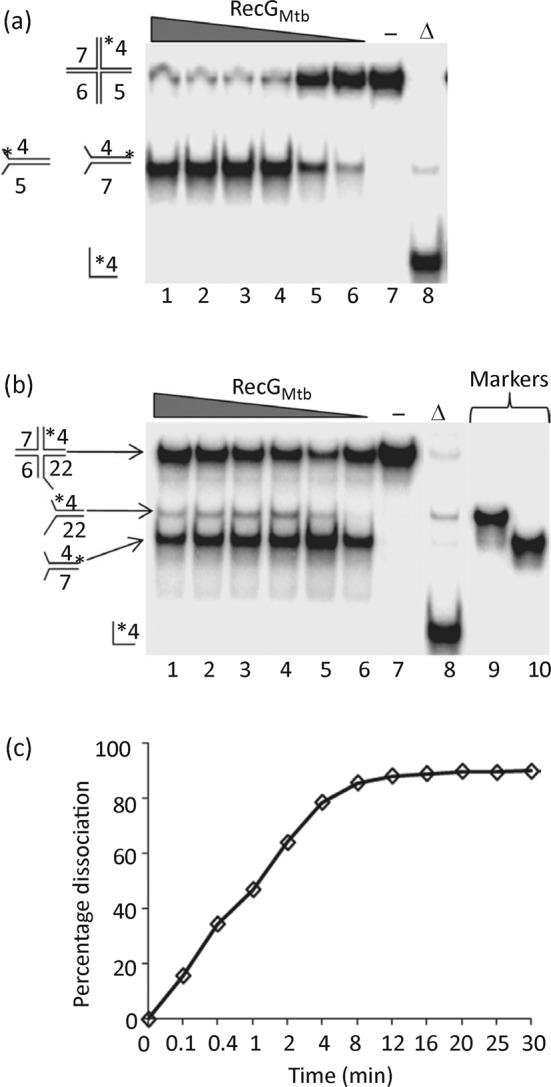
RecG_Mtb_ dissociates HJ substrates to flayed duplexes. (a) Titration of branch migration activity using 250, 200, 150, 100, 50 and 25 nM RecG_Mtb_ (lanes 1–6, respectively) and 0.5 nM HJ substrate. Lanes: (−), reaction lacking RecG_Mtb_; (Δ), heat-denatured substrate. (b) Direction of branch migration of HJs. Reactions were conducted in the same way as in (a) except that a modified HJ substrate in which oligonucleotide 5 was replaced with oligonucleotide 22 was used (Tables 1 and 2). Lanes 9 and 10 show size markers for the expected branch migration products. (c) Time course of dissociation of HJ substrate by RecG_Mtb_. The helicase reaction (140 µl) was carried out using 150 nM RecG_Mtb_ and 0.5 nM HJ substrate. Data in (c) are the average of two independent experiments.

RecG_Mtb_ also unwound an HJ substrate with a 12 bp central homologous ‘movable core’, producing flayed duplexes (Fig. 4a). To determine the direction of RecG_Mtb_-mediated branch migration of the HJ, RecG_Mtb_ was challenged with an HJ substrate containing a 16 nt extension on one of the four duplex arms (Fig. 4b). Interestingly, RecG_Mtb_ appeared to drive branch migration bidirectionally. The time course of HJ substrate unwinding indicated that nearly half (47 %) of the substrate was converted to flayed duplex by RecG_Mtb_ within 1 min (Fig. 4c).

**Fig. 5.**
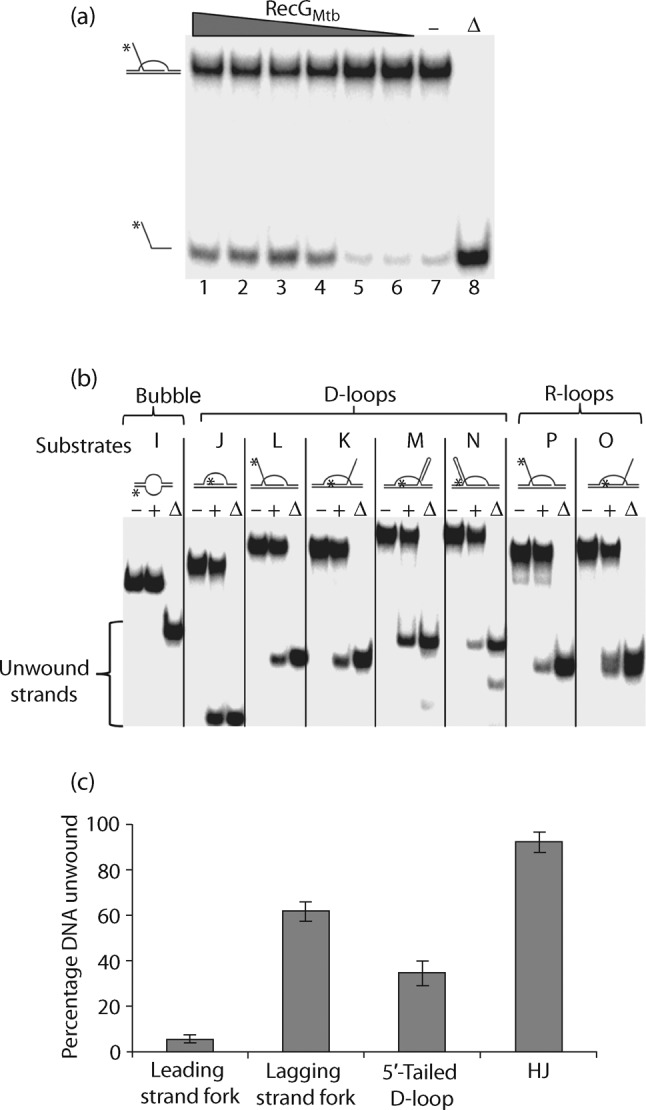
RecG_Mtb_ unwinds the invading strands from a variety of synthetic D- and R-loop structures. (a) Titration of helicase activity using 250, 200, 150, 100, 50 and 25 nM RecG_Mtb_ (lanes 1–6, respectively) and 0.5 nM 5′-tailed D-loop (substrate L, Table 2). (b) Helicase assay using 150 nM RecG_Mtb_ and 0.5 nM of the indicated substrates (substrates I–P, Table 2). Lanes: (−), absence of RecG_Mtb_; (+), presence of RecG_Mtb_; (Δ), heat-denatured samples. (c) RecG_Mtb_ has a predilection for dissociating HJ compared with D-loop and replication fork substrates. The helicase assay was conducted using 150 nM RecG_Mtb_ and 0.5 nM of the indicated DNA substrates. Data are means±sd from three independent experiments.

RecG_Mtb_ also unwound a variety of synthetic D- and R-loop structures. These included D-loops without tail (substrate J), D-loops with 5′ or 3′ tails (substrates L and K, respectively), D-loops with a hairpin end at the 5′ or 3′ tail (substrates N and M, respectively), and R-loops with 5′ and 3′ (2′-*O*-methyl-RNA) tails (substrates P and O, respectively) (Fig. 5a, b). The 2′-*O*-methyl modification was introduced to protect the RNA against nuclease degradation (Inoue *et al.*, 1987). The ability of RecG_Mtb_ to unwind D- and R-loops regardless of the absence or presence of a tail suggests that RecG_Mtb_ may preferentially interact with such DNA structures at the junction. RecG_Mtb_ might also pull one side or a segment of the duplex arm of the D- or R-loop structure through the wedge-containing domain, thereby stripping the invading strand off the loop structure, as previously proposed for unwinding of the lagging strand from a partial replication fork (Rudolph *et al*., 2010b; Singleton *et al*., 2001).

We determined the efficiency with which RecG_Mtb_ unwound HJ, replication fork and D-loop substrates, and identified HJ as the preferred DNA substrate of RecG_Mtb_ helicase, followed by the lagging strand replication fork (Fig. 5c). These two DNA substrates were also preferentially bound by RecG_Mtb_ (Fig. 2c). On the other hand, RecG_Mtb_ did not unwind blunt-end DNA duplex (Fig. S2a), 3′- or 5′-tailed DNA duplex (Fig. S2b, c), or a bubble substrate (Fig. 5b).

### Divalent metal ion and nucleotide requirements for RecG_Mtb_ unwinding activity

**Fig. 6.**
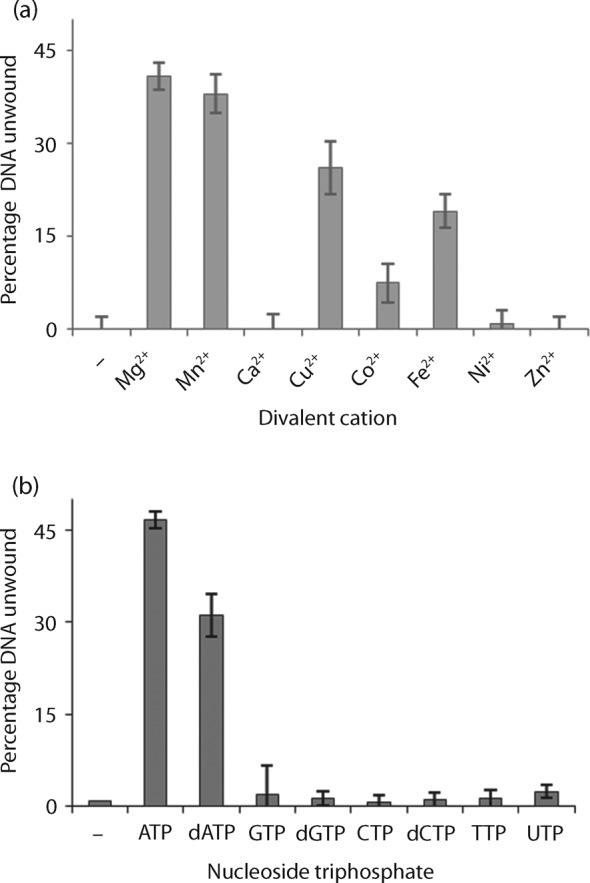
Divalent cation and NTP/dNTP specificity of RecG_Mtb_ for its helicase activity. (a) Effects of divalent metal cofactors on RecG_Mtb_ helicase activity. The helicase assay was performed in the presence of 2 mM of the indicated divalent metal cofactor, RecG_Mtb_ (150 nM) and lagging strand replication fork substrate (0.5 nM). (b) Fuel specificity for RecG_Mtb_ helicase activity. The helicase assay was performed in the presence of 2 mM of the indicated NTP/dNTP, RecG_Mtb_ (150 nM) and lagging strand replication fork substrate (0.5 nM). Data are means±sd from four independent experiments.

The RecG_Mtb_ helicase was active in the presence of magnesium, manganese, copper, iron or cobalt ions, but completely inactive in the reactions lacking divalent cations (Fig. 6a). No significant difference was observed in RecG_Mtb_ unwinding activity in the presence of Mn^2+^ or Mg^2+^ (*P* = 0.125). RecG_Mtb_ unwound DNA substrates in the presence of ATP or dATP, but was inactive in the presence of other NTP/dNTPs (Fig. 6b). RecG_Mtb_ unwinding activity was significantly higher in the presence of ATP than dATP (*P* = 0.023). Furthermore, ADP and the slowly hydrolysable ATP analogue ATPγS did not support the unwinding activity of RecG_Mtb_ (Fig. S3).

### RecG_Mtb_ is a DNA-dependent ATPase

**Fig. 7.**
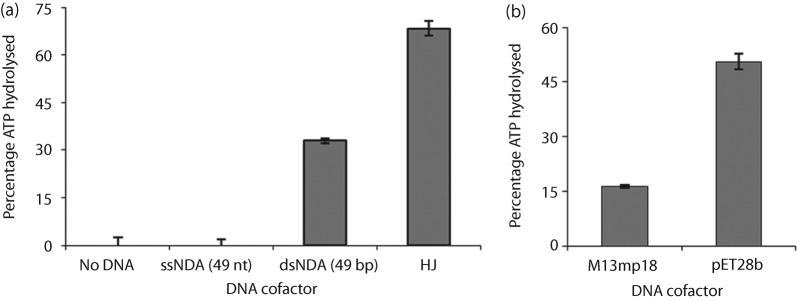
ATP hydrolysis by RecG_Mtb_. (a) Effects of DNA cofactors on RecG_Mtb_ ATPase activity. The assays were conducted in the presence of 300 nM RecG_Mtb_ and 50 nM of the indicated unlabelled DNA cofactor (a) or 10 nM of the indicated circular plasmid DNA (b). Plasmid pET28b was purified using Qiagen Midi kits, whereas M13mp18 (NEB) was used as obtained. Data are means±sd from at least three independent experiments.

The ATPase activity of RecG_Mtb_ was measured in the presence of 50 nM ssDNA (49 nt), dsDNA (49 bp) or an HJ substrate (assembled from four ~49 nt oligos). The efficiency of ATP hydrolysis was 68, 32 and 0 % in the presence of HJ, dsDNA and ssDNA, respectively (Fig. 7a). Moreover, no ATP hydrolysis was observed in the reactions containing 20–100 nt poly(dT) (data not shown). We also tested ATP hydrolysis in the presence of 10 nM circular DNA cofactors, M13mp18 (ssDNA) or pET28b (dsDNA); pET28b (51 %) stimulated threefold higher ATP hydrolysis than M13mp18 (16 %) (Fig. 7b). To avoid intramolecular duplex formation, all the ssDNA cofactors tested in this study were heated to 95 °C for 5 min immediately before use.

Steady-state kinetic analysis of RecG_Mtb_ ATPase activity was performed by titrating ATP in the presence of saturating circular dsDNA (pET28b). Under these conditions, ATP hydrolysis was linear for at least 15 min (data not shown). Michaelis–Menten (hyperbolic) curve-fitting analysis showed that the mean (±se) *V*_max_, *K*_m_ and *K*_cat_ of RecG_Mtb_ were 477 (±27) µM min^−1^, 202 (±35) µM and 3180 (±59) min^−1^, respectively. Using the Hill (allosteric sigmoidal) equation to model the data, the mean (±se) Hill coefficient (*H*) obtained was 1.0±0.3, which suggests a monomeric state during ATP hydrolysis.

## Discussion

The *M. tuberculosis* genome is susceptible to the effects of genotoxic and general cellular stress, including nitrosative and oxidative damage to DNA, RNA and other biomolecules (Stallings & Glickman, 2010; Warner & Mizrahi, 2006), owing to the harsh internal environment, the human macrophage, in which it usually resides. Mechanisms that promote genome maintenance and function are likely to be essential for *M. tuberculosis* survival and virulence, because persistent unrepaired DNA damage can completely block replication of the genome (Masai *et al.*, 2010; McGlynn & Lloyd, 2001; Mirkin & Mirkin, 2007). RecG is an important enzyme widely thought to play a role in remodelling replication forks stalled at DNA lesions, mediating replication restart via fork regression (McGlynn & Lloyd, 2001, 2002). In the present study, the biochemical activities of RecG_Mtb_ are characterized, providing considerable insight into the potential role(s) of RecG in DNA/nucleic acid metabolism in an intracellular pathogen.

This study demonstrated that RecG_Mtb_ binds and unwinds a variety of DNA substrates that mimic intermediates in DNA replication, recombination and repair, like its *E. coli* orthologue. Among the substrates examined here, RecG_Mtb_ had the highest affinity for HJ, while 3′- and 5′-overhang DNA substrates and blunt-end duplex DNA substrates were bound very poorly (Table 2). In general, the binding and unwinding activity of RecG_Mtb_ required protein concentrations that were considerably higher than those described for *E. coli* RecG. Whereas *E. coli* RecG shifted an HJ substrate at protein concentrations of 0.1 nM (Briggs *et al.*, 2005), the same shift was only observed with 0.25 µM RecG_Mtb_. This might be due to suboptimal assay conditions. Generally, *M. tuberculosis* helicases exerted their activities at concentrations >100 nM (Balasingham *et al.*, 2012; Biswas *et al.*, 2009; Curti *et al.*, 2007).

Unexpectedly, RecG_Mtb_ had very low affinity for poly(dA), and much higher affinity for poly(dT), poly(dG) and poly(dC). Generally, RecG_Mtb_ and other superfamily 2 helicases make extensive contact with the sugar-phosphate DNA backbone and this interface is the dominant functional mode of interaction (Büttner *et al.*, 2007; Kim *et al.*, 1998; Pyle, 2008; Singleton *et al.*, 2001). DNA helicases also tend to exhibit low DNA sequence specificity, presumably because higher sequence specificity might hinder the translocation and/or processivity of the helicase (Rocak & Linder, 2004; Tanner & Linder, 2001; Tuteja & Tuteja, 2004). However, a recent report has revealed that *Vaccinia* viral helicase NPH-II, a superfamily 2 helicase, favours purine-rich over pyrimidine-rich dsDNA helicase substrates (Taylor *et al.*, 2010). The sequence bias of RecG_Mtb_ reported here might be an intrinsic property of this helicase; however, this conclusion is preliminary and requires additional investigation.

Notably, another interesting observation reported here is that the affinity of RecG_Mtb_ for ssDNA and dsDNA is length-dependent. RecG_Mtb_ had higher affinity for longer oligomers (≥40 nt) than for shorter oligomers (20 nt). This suggests that 20 nt/bp DNA substrates are not long enough to form a stable complex with RecG_Mtb_. The relatively more stable binding of RecG_Mtb_ ≥40 nt poly(dT) is consistent with the site size determined for *E. coli* RecG for poly(dT), 36 nt (Slocum *et al.*, 2007). RecG_Mtb_ may be recruited to stalled replication forks via an interaction with ssDNA binding protein (SSB) (as for *E. coli* RecG) (Buss *et al.*, 2008; Lecointe *et al.*, 2007; Liu *et al.*, 2011). Nevertheless, the observed interaction between RecG_Mtb_ and ssDNA may represent an alternative mechanism for targeting RecG to stalled replication forks or other branched DNA substrates.

Even though RecG_Mtb_ binds a variety of DNA substrates, including ssDNA, it only unwinds branched DNA substrates, including HJs, replication forks, and D- and R-loops. This specificity suggested a potential involvement of RecG_Mtb_ in multiple DNA metabolic pathways of mycobacteria, including recombination, replication and repair. The relatively high affinity of RecG_Mtb_ for the HJ and the fact that HJs maximally stimulate RecG_Mtb_ ATPase activity indicate that HJs could be a relevant *in vivo* substrate for RecG_Mtb_.

RecG_Mtb_ required Mg^2+^ for its optimal activity, although unwinding activity is also supported by Mn^2+^, Cu^2+^, Co^2+^ and Fe^2+^. A similar observation was also reported for another mycobacterial helicase, UvrD (Curti *et al.*, 2007). This property, shared by these two *M. tuberculosis* helicases, could contribute to the pathogenicity of *M. tuberculosis*, because Mg^2+^ is scarce in the phagosomes of macrophages (Groisman, 1998). The observation that the ATPase activity of RecG_Mtb_ was stimulated to a greater extent in the presence of dsDNA than of ssDNA suggests that the enzyme may translocate on dsDNA, as does *E. coli* RecG (Mahdi *et al.*, 2003).

Evidence shows that expression of *recG* in *M. tuberculosis* is upregulated in infected human cells and mouse macrophages, suggesting that RecG may actively promote virulence and/or pathogenicity during infection of mammalian cells (Davis & Forse, 2009; Rachman *et al.*, 2006; Schnappinger *et al.*, 2003). It is also interesting that *recG* is conserved in the related human pathogen *Mycobacterium leprae*, in which there is an extreme case of reductive evolution (Vissa & Brennan, 2001). This suggests a potentially important metabolic role for RecG in other mycobacteria also. The genotoxic stress that *M. tuberculosis* encounters inside the macrophage with reactive nitrogen and oxygen species is very different from that to which *E. coli* cells are exposed in their various environmental niches. Thus, the metabolic conditions inside an intracellular pathogen such as *M. tuberculosis* might be considerably different from those of *E. coli* cells and other model species. This is exemplified by the facts that the genome of *M. tuberculosis* comprises an unusually high number of genes involved in lipid metabolism (>233), that its genome has a high G+C content (Cole *et al.*, 1998), and that there is a lack of MutS-based mismatch repair in *M. tuberculosis* (Mizrahi & Andersen, 1998). The existence of a non-homologous end-joining pathway (Della *et al.*, 2004) as well as an alternative regulatory mechanism for DNA damage-inducible genes (Davis *et al.*, 2002) have also been indicated in *M. tuberculosis*. A recent study further has indicated that RuvAB of *M. tuberculosis*, unlike *E. coli* RuvAB, can convert replication forks to HJs (Khanduja & Muniyappa, 2012). Moreover, biochemical characterization of DNA repair components indicates that oxidative DNA glycosylases of *M. tuberculosis* exhibit substrate preferences different from their *E. coli* counterparts (Guo *et al.*, 2010). Taken together, these findings suggest that the DNA metabolism of *M. tuberculosis* might differ considerably from that of *E. coli*.

### Conclusions

The novel findings presented here that RecG exists in vascular plants and algae, in addition to eubacteria, and that RecG_Mtb_ preferentially binds relatively long ssDNA, exhibiting a higher affinity for poly(dT), poly(dG) and poly(dC) than for poly(dA), shed new light on the occurrence and role of RecG in nature. Furthermore, the finding that the preferred helicase substrate for RecG_Mtb_ is HJ, a key intermediate in DNA repair, recombination and replication fork restart (Kepple *et al.*, 2005; Liu & West, 2004), may suggest that RecG_Mtb_ is preferentially involved in such processes *in vivo*. However, future studies involving *M. tuberculosis recG*-null mutants are needed to clarify the precise role of RecG in the DNA metabolism, survival, fitness and virulence of *M. tuberculosis* and possibly of other related mycobacteria.
